# Marine Carotenoid Fucoxanthin Possesses Anti-Metastasis Activity: Molecular Evidence

**DOI:** 10.3390/md17060338

**Published:** 2019-06-05

**Authors:** Sukant Garg, Sajal Afzal, Ahmed Elwakeel, Damini Sharma, Navaneethan Radhakrishnan, Jaspreet Kaur Dhanjal, Durai Sundar, Sunil C. Kaul, Renu Wadhwa

**Affiliations:** 1 DAILAB, DBT-AIST International Center for Translational & Environmental Research (DAICENTER), National Institute of Advanced Industrial Science & Technology (AIST), Tsukuba 305-8565, Japan; sukantgarg@gmail.com (S.G.); sajal.afzal@aist.go.jp (S.A.); ahmed.elwakeel@aist.go.jp (A.E.); sharmadamini823@gmail.com (D.S.); jaspreetk.dhanjal@aist.go.jp (J.K.D.); 2 School of Integrative & Global Majors, University of Tsukuba, Tsukuba 305-8577, Japan; 3 DAILAB, Department of Biochemical Engineering & Biotechnology, Indian Institute of Technology (IIT) Delhi, Hauz Khas, New Delhi 110-016, India; navaneethanbio@gmail.com (N.R.); sundar@dbeb.iitd.ac.in (D.S.)

**Keywords:** fucoxanthin, cancer, p53–mortalin interaction, abrogation, growth arrest, therapy

## Abstract

Fucoxanthin is commonly found in marine organisms; however, to date, it has been one of the scarcely explored natural compounds. We investigated its activities in human cancer cell culture-based viability, migration, and molecular assays, and found that it possesses strong anticancer and anti-metastatic activities that work irrespective of the p53 status of cancer cells. In our experiments, fucoxanthin caused the transcriptional suppression of mortalin. Cell phenotype-driven molecular analyses on control and treated cells demonstrated that fucoxanthin caused a decrease in hallmark proteins associated with cell proliferation, survival, and the metastatic spread of cancer cells at doses that were relatively safe to the normal cells. The data suggested that the cancer therapy regimen may benefit from the recruitment of fucoxanthin; hence, it warrants further attention for basic mechanistic studies as well as drug development.

## 1. Introduction

Fucoxanthin is a pigment that is widely distributed in brown algae (especially *Undaria pinnatifida*) and diatoms, and has a uniquely interesting xanthophyll chemical structure consisting of an allenic bond, nine unconjugated double bonds, a 5,6-monoepoxide moiety, and a few other oxygenic functional groups [1]. Known widely for its anti-stress and antioxidant properties, it was reported to be tolerable in mice up to the daily dose of 1 g/kg body weight for 30 days [2]. Its role as an anticancer molecule has been explored. At lower doses, it was shown to upregulate Bcl-xL and p21^WAF1/Cip1^, and inhibit JAK/STAT and cyclin D, leading to the growth arrest [3,4,5] in multiple cancer cell lines. Growth arrest has most commonly been shown in the G_1_ phase [6,7] in vitro and in vivo. Relatively high doses of fucoxanthin caused the cleavage of caspases (3, 7, 8, and 9) and PARP via the formation of reactive oxygen species (ROS), leading to apoptosis in HL-60 and HTLV-1-infected T-cell leukemia cells, and primary effusion lymphomas [4,8,9]. It has been shown to inhibit the JAK/STAT and ERK/PI3K/AKT pathways, and angiogenesis in MGC-803 gastric cancer, HepG2 hepatic cancer, and HUVECs, respectively [3,10,11]. It suppressed the formation of lamellipodia and the metastatic characteristics of the highly invasive B16-F10 murine melanoma cells via suppression of the CD44/CXCR4 at the mRNA and MMP9 at the protein levels [12]. In colon cancer cells, fucoxanthin caused cell cycle arrest in G_1_ phase via the activation of p21^WAF1/Cip1^ and p27^Kip1^ [5] and apoptosis via the suppression of Bcl-2 [13]. Furthermore, it has also been shown to have cancer chemopreventive potential for colon cancer [14,15]. In the recent study, we found that the low non-toxic doses of fucoxanthin triggered differentiation in C6 glioma cells [16].

Cancer is a highly complex syndrome consisting of the loss of cell proliferation control, cellular homeostasis, physiological disruption, and its metastatic spread. It has been termed as a rising epidemic and is known to claim millions of lives each year globally [17]. Metastasis is one of the major underlying mechanisms of circulating cancers [18]. Although little understood so far, the strongest and the most aggressive forms of cancer cell phenotypes have been shown to infiltrate distant tissue and evade immune responses, adapt to supportive niches, survive as latent tumor-initiating seeds, and replace the tumor microenvironment for metastatic colonization. Some of the major factors that drive metastasis include the epithelial to mesenchymal transformation (EMT), which is regulated tightly by the activation of Wnt/β-catenin signaling. Activated Wnt communicates with the frizzled receptor located on the cell membrane, leading to the inactivation and dissociation of the Axin/APC/CK1/GSK3β/β-catenin complex. Then, excessively unbound β-catenin translocates to the nucleus and complexes with TCF-CBP to activate downstream cell cycle-promoting proteins, including SMADs, RB, and cyclins. Major proteins participating in the EMT include the mesenchymal structural regulator vimentin, adhesion/migration marker fibronectin, and extracellular matrix degrading proteins MMPs. Other pathways such as MAPK/ERK and JAK/STAT signaling also finely regulate cell survival and proliferation, making cancer cells autonomous and independent of the external stimuli and growth inhibitory signals. In all, these phenomena at large constitutively lead to aggressive disease and poor clinical prognosis. Importantly, these processes are suppressed and limited by the activation and overexpression of the tumor suppressor *TP53* gene and p53 protein. The latter plays a major role in the activation of pathogen recognition, DNA repair, growth arrest, apoptosis, and senescence [19,20], and is functionally inactivated or lost in a large majority of cancers [21,22]. Besides mutations, p53 is known to be inactivated via post-translational modifications and cytoplasmic sequestration [23,24,25]. Wild-type p53 is also called the guardian of genome and tightly regulates the cell cycle, replicative senescence, and DNA damage response. The mutations and/or its functional inactivation of p53 have been shown to contribute to immortalization and carcinogenesis by multiple pathways [26]; in turn, p53 gets regulated by several other proteins including HDM2, ARF, p21, ATM/ATR, AKT, Beclin1, Puma, and Noxa [26]. It has been shown that the stress chaperone mortalin interacts with p53 and inactivates its transcriptional activation [27]. This interaction takes place between the C-terminal amino acid (a. a.) residues 312–352 of p53 and a. a. residues 253–282 of mortalin. The latter is enriched in cancer cells, and the abrogation of mortalin–p53 interactions have been shown to reactivate p53, yielding growth arrest/apoptosis [27,28,29]. Mortalin has also been shown to play a role in EMT transition [30] and cancer cell stemness [31,32,33]. In view of the above information, we investigated the potential of fucoxanthin on mortalin–p53 interaction and the subsequent effect on cell migration and metastasis signaling. By bioinformatics and molecular docking analysis, we found that fucoxanthin has the potential to bind to p53, but not mortalin. However, it downregulated mortalin at the transcriptional level and yielded growth arrest/apoptosis. Low non-toxic doses of fucoxanthin caused a delay in cell migration and invasion in cancer cells, irrespective of their p53 status. The results proposed that in spite of being light and heat sensitive, fucoxanthin has potential as a natural anticancer and anti-metastasis compound, which warrants not only the basic molecular studies, but also the attention of the pharmaceutical industry.

## 2. Results

### 2.1. Fucoxanthin Caused Activation of P53 Function in Cancer Cells

**Figure 1:**
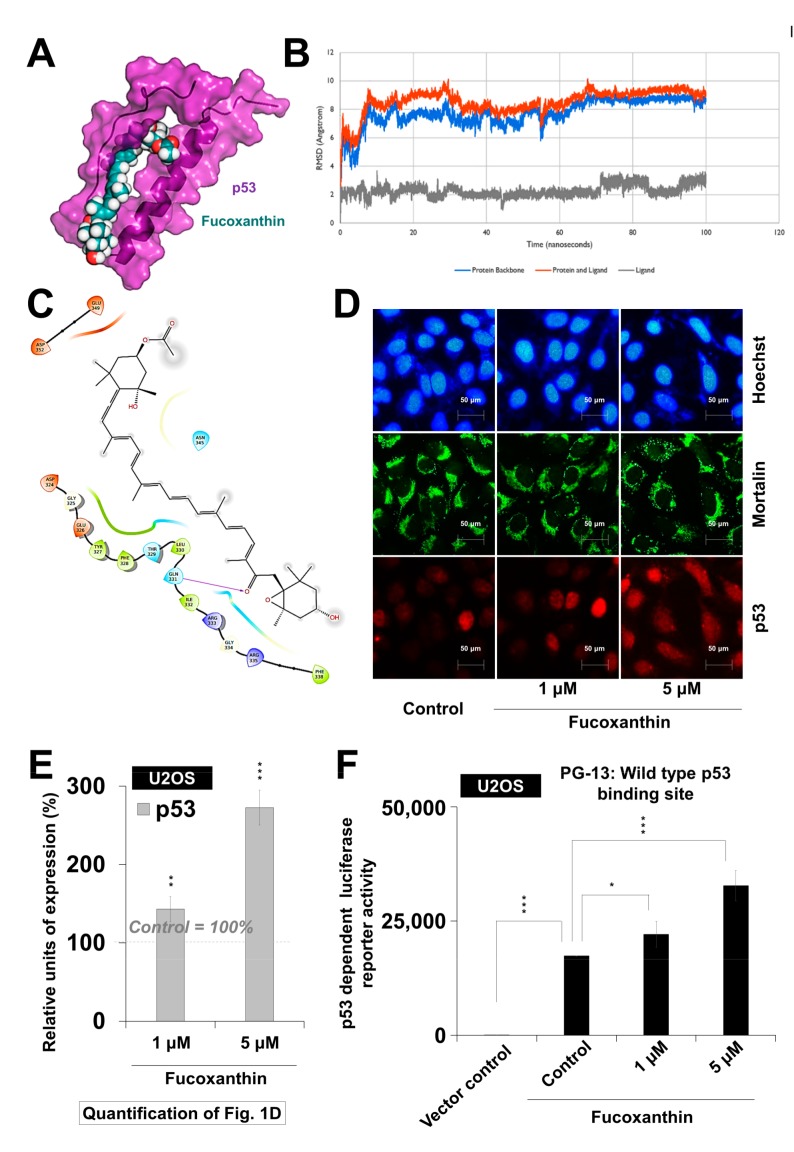
Activation of wild-type p53 by fucoxanthin. (**A**) Graphical representation of fucoxanthin docked into the mortalin binding site of p53; (**B**) RMSD of structures in p53–fucoxanthin complex showing the formation of stable hydrogen bond interactions; (**C*)*** In silico molecular docking analysis showing the capability of fucoxanthin to interact with p53; (**D**) Double immunostaining for mortalin and p53 in control and treated U2OS (wild-type p53) cells; (**E**) Quantification of nuclear p53 is shown; (**F**). Wild-type p53 driven luciferase reporter **(**PG-13-Luc) assay showing the activation of wild-type p53 in fucoxanthin-treated cells. * *p* < 0.05, ** *p* < 0.01, *** *p* < 0.001.

Based on earlier reports, the small molecules that could abrogate p53–mortalin interaction cause the growth arrest of cancer cells [27,28]. So, we performed in silico analyses to examine the interaction of fucoxanthin with the p53–mortalin complex. Molecular docking analyses revealed that fucoxanthin could bind to p53 (Figure 1A), but not to mortalin. It formed interactions with p53 (docking score −2.768 kcal/mol) involving the amino acid residues from Asp 324 to Asp 352, and was found to be stably interacting at the docked site in p53 throughout the 100-ns molecular dynamics simulation run. Fucoxanthin-bound p53 deviated from its initial structure in the first 10 ns, but acquired a quite stable confirmation thereafter (Figure 1B). Despite these changes in the protein backbone, no significant change was observed in the binding of fucoxanthin (Figure 1B). The molecular interactions between p53 and fucoxanthin were mainly hydrophobic in nature, with only one hydrogen bond involving Gln 331 (Figure 1C). The data suggested that fucoxanthin might act as a competitive inhibitor by preventing the interaction of mortalin with p53, setting p53 free to migrate into the nucleus, and performing its transcriptional activation function. To validate this hypothesis, we examined the activity of p53 in control and fucoxanthin-treated cells by examining the (i) nuclear translocation of p53 (Figure 1D,E) and (ii) the transcriptional activation function of wild-type p53 using the PG-13Luc reporter (Figure 1F). These assays supported the nuclear translocation of p53 in fucoxanthin-treated cells, as was predicted by computational analysis. In wild-type p53-dependent luciferase reporter assays, it was confirmed that fucoxanthin led to the activation of wild type p53 (Figure 1F). These results showed that fucoxanthin caused the abrogation of mortalin–p53 interactions, resulting in nuclear translocation and the activation of p53 in cancer cells. Since p53–mortalin interactions are unique to cancer cells [27,29], we predicted that fucoxanthin may be selectively toxic to cancer cells.

### 2.2. Fucoxanthin Was Selectively Toxic to Cancer Cells

**Figure 2:**
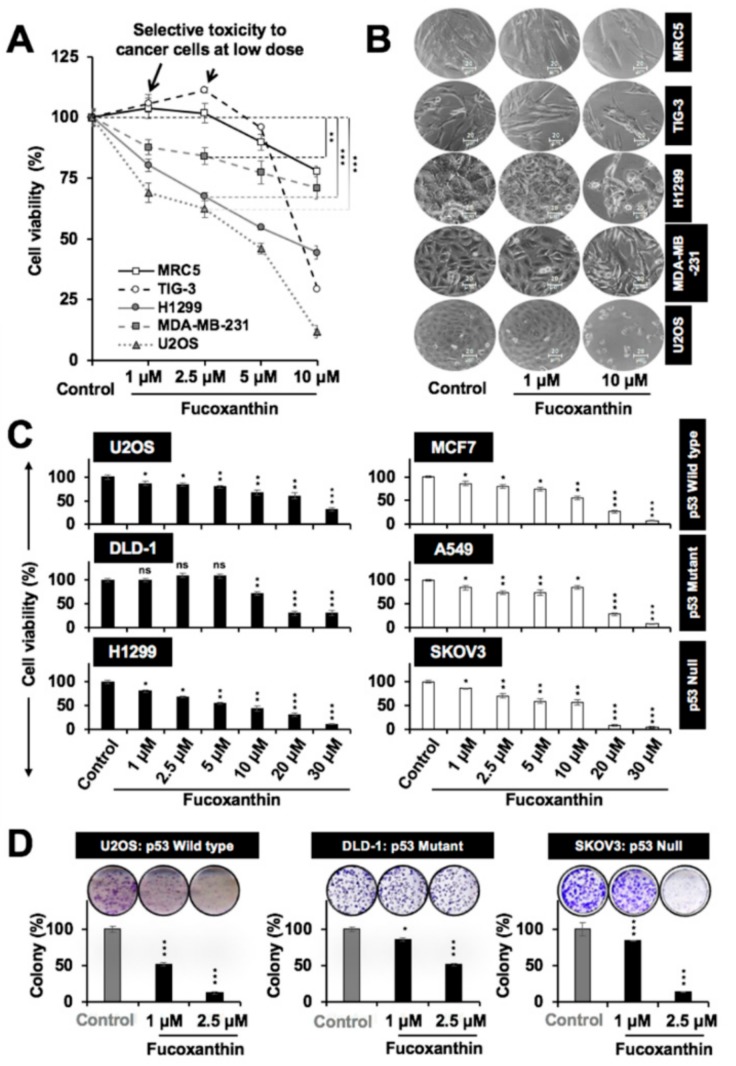
Cytotoxicity of fucoxanthin to different cancer cells. (**A**) Cell viability assays showed the dose-dependent cytotoxicity of fucoxanthin to various cancer cells. Low doses of 1.0 and 2.5 M showed selective toxicity in the cancer cells; (**B**) Phase contrast images of cells treated with fucoxanthin for 48 h showing dose-dependent cytotoxicity; (**C**) Dose-dependent cytotoxicity of fucoxanthin in the cancer cells with wild type (U2OS and MCF7), mutant (DLD-1 and A549) and null (H1299 and SKOV3) p53 status; (**D**) Dose-dependent inhibition of the colony-forming potential of the cells with variable p53 status by the long-term treatment of fucoxanthin. * *p* < 0.05, ** *p* < 0.01, *** *p* < 0.001, ns = not significant.

Next, we determined the dose-dependent cytotoxicity of fucoxanthin in a variety of cancer cells. Normal human fibroblasts (MRC5 and TIG-3) were used as controls. We found that it was selectively toxic to cancer cells with no effect on normal cells up to a dose of 2.5 µM (48-h treatment), as shown in the growth curves (Figure 2A) and the cell morphology (Figure 2B). Since the loss of p53 function (by mutations/deletions/interactions with other proteins) is an established hallmark of cancer cells, we next examined the cytotoxic response of cancer cells with variable p53 status, and found that fucoxanthin was cytotoxic to these cancer cells, irrespective of their p53 status. Representative cells with wild-type p53 (U2OS and MCF7), mutant p53 (DLD-1, A549, and MDA-MB-231) and null p53 (H1299 and SKOV3) showed similar dose-dependent responses (Figure 2C and data not shown). The IC_50_ doses of fucoxanthin in all of the examined cells are tabulated in Figure S1A. During the course of these experiments, we observed that the cytotoxicity of fucoxanthin was affected by its storage condition. Therefore, we investigated its physical characteristics when exposed to light and temperature (Supplementary Figure S2). Irrespective of the absolute values for cytotoxicity in each experiment, a similar trend was observed in different experiments. Quantitative Cell Viability (QCV) assay [34] (long-term) confirmed that fucoxanthin caused the growth inhibition of cancer cells, irrespective of their p53 status (Figure 2D and Figure S1B). Taken together, these data suggested that fucoxanthin-mediated cytotoxicity to cancer cells may also be mediated by mechanisms independent to the abrogation of mortalin–p53 interactions. 

### 2.3. Fucoxanthin Caused Downregulation of Mortalin Expression

**Figure 3:**
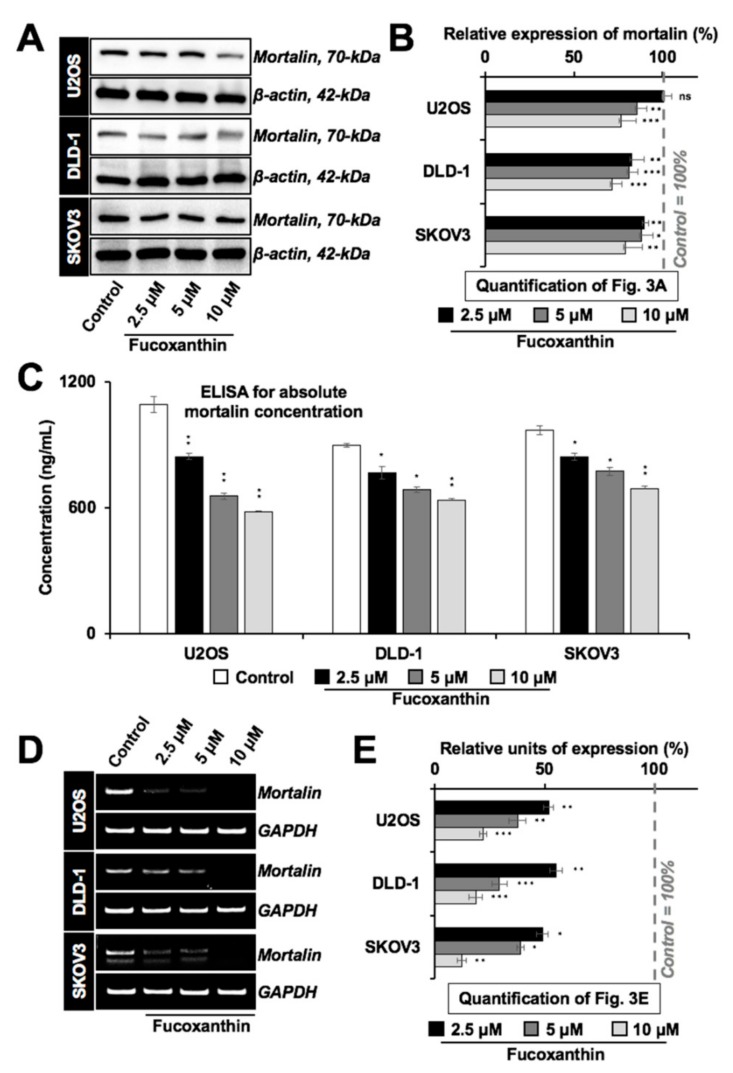
Fucoxanthin caused transcriptional downregulation of mortalin. (**A**) Western blot showing dose-dependent decrease in mortalin in fucoxanthin-treated cells with wild type p53 (U2OS), mutant p53 (DLD-1), and null p53 (SKOV3) status; (**B**) Histogram shows the quantification of the mortalin level of expression shown in Figure 3A; (**C**) Histogram showing mortalin concentration in fucoxanthin-treated p53 wild type (U2OS), mutant (DLD-1), and null (SKOV3) cells determined by sandwich ELISA using anti-mortalin antibody; (**D**) RT-PCR analysis showing dose-dependent transcriptional downregulation of mortalin in fucoxanthin-treated p53 wild type (U2OS), mutant (DLD-1), and null (SKOV3) cells. (**E**) Histogram shows the quantification of Figure 3D. * *p* < 0.05, ** *p* < 0.01, *** *p* < 0.001, ns = not significant.

We next performed Western blotting for mortalin in cancer cell lines with variable p53 status. As shown in Figure 3A,B, fucoxanthin suppressed the expression of mortalin in a dose-dependent manner independent of the p53 status. The results were confirmed by mortalin ELISA (Figure 3C) and immunostaining (data not shown). We next performed RT-PCR (Figure 3D,E) that revealed the downregulation of mortalin at the transcript level in fucoxanthin-treated cells.

### 2.4. Subtoxic Dose of Fucoxanthin Possesses Anti-Metastasis Potential

**Figure 4:**
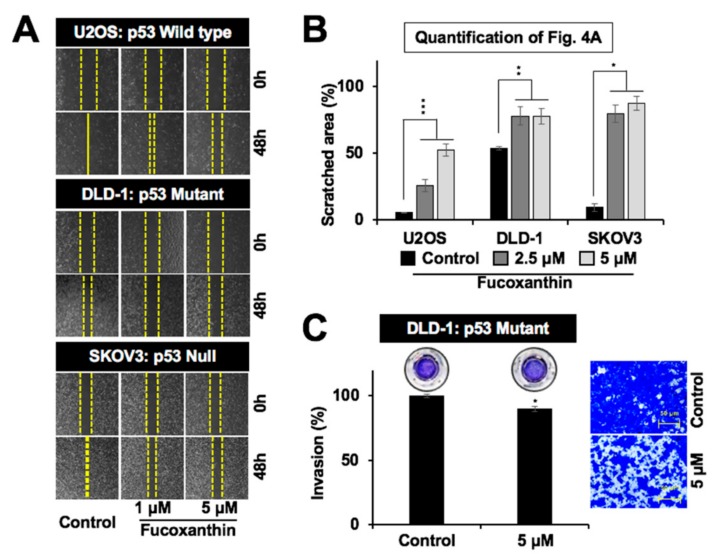
Low doses of fucoxanthin caused the inhibition of migration and invasion of cancer cells with varying p53 status. (**A**) Wound-scratch migration assay images showing the inhibition of the migration capacity of p53 wild-type (U2OS), mutant (DLD-1), and null (SKOV3) cells treated with subtoxic doses of fucoxanthin; (**B**) Quantification of wound scratch migration from Figure 4A; (**C**) Matrigel invasion assay images and quantification showing the inhibition of the invasiveness of p53 mutant (DLD-1) cells treated with a subtoxic dose of fucoxanthin. * *p* < 0.05, ** *p* < 0.01, *** *p* < 0.001.

**Figure 5:**
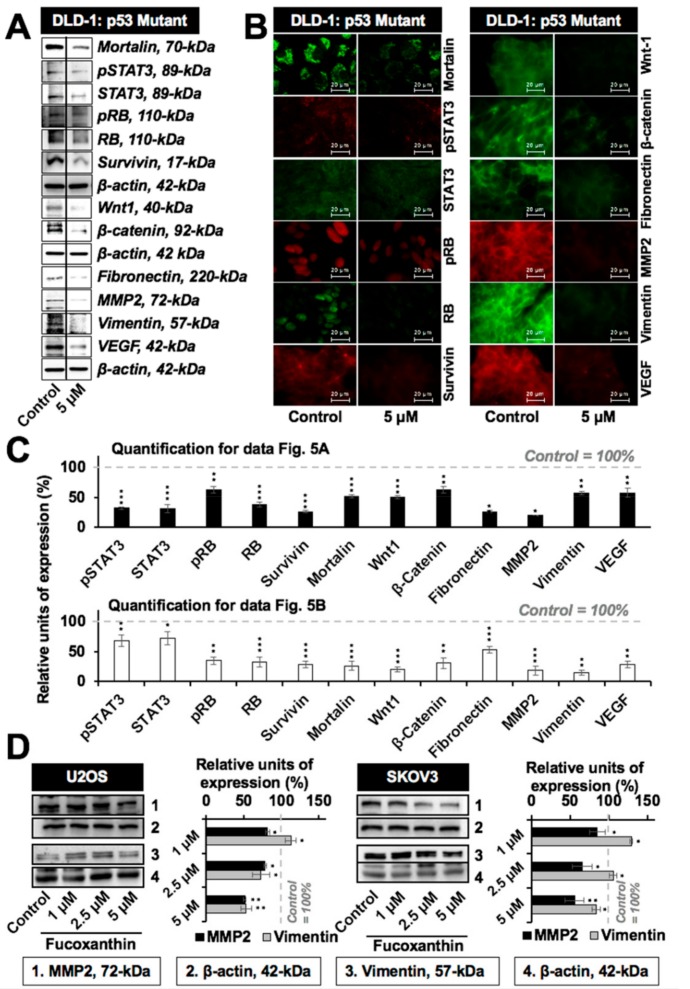
Low doses of fucoxanthin caused the inhibition of key regulators of metastasis. (**A**) Western blotting showing the downregulation of metastasis-associated and proliferation-associated proteins in DLD-1 (mutant p53) cells treated with a subtoxic dose of fucoxanthin; (**B**) Immunostaining of DLD-1 cells showing the downregulation of metastasis-associated and proliferation-associated proteins subsequent to fucoxanthin treatment; (**C**) Quantification of Western blotting and immunostaining results from Figure 5A,B normalized to their respective controls; (**D**) Western blotting and its quantification showing the downregulation of major metastasis regulatory proteins (MMP2 and vimentin) in p53 wild-type (U2OS) and null (SKOV3) cells treated with a subtoxic dose of fucoxanthin. * *p* < 0.05, ** *p* < 0.01, *** *p* < 0.001.

In light of the role of mortalin in cell migration and metastasis [30,31,32,33], we next examined the effect of fucoxanthin on these characteristics of cancer cells. As shown in Figure 4A,B, the subtoxic doses (up to 5 µM) of fucoxanthin caused a significant delay in the migration of cells with variable p53 status. These results were further supported by a cell invasion assay that showed the significant anti-invasive potential of fucoxanthin (Figure 4C and data not shown). Biochemical analysis revealed that the subtoxic (5 µM, 48 h) treatment of fucoxanthin in DLD-1 cells caused the significant downregulation of mortalin. Furthermore, proliferation-associated proteins (STAT3, pSTAT3, RB, and pRB), survival protein (survivin), stemness-related proteins (Wnt-1 and β-catenin), EMT markers (fibronectin, MMP2, and vimentin), and angiogenesis-factor (VEGF) showed a decrease in both Western blotting and immunostaining assays (Figure 5A–C). Of note, the expression of EMT markers MMP2 and vimentin was also found to be dose-dependently and significantly downregulated in p53 wild-type U2OS and p53 null SKOV3 cells (Figure 5D). These results advocated the anti-metastatic potential of fucoxanthin.

## 3. Discussion

Infinite proliferation, survival, disrupted physiology, autonomous growth in colonies, and gain in migratory capacity are the hallmark characteristics of cancer [35]. Interactions between cancer tissues, its associated stroma, and its microenvironment represent a powerful relationship that determines disease initiation, progression, and prognosis. Depending upon the type, origin, and time of colonization in their niche, cells transform into malignant subtypes and metastasize to distant sites [18,36]. Various mechanisms such as the rapid proliferation of the survivors, EMT, and angiogenesis play crucial roles in metastasis development. The crucial factor in the development of anticancer drugs is to counter the acquired migration in cancer cells. The conventional chemotherapy regime largely depends on the synthetic molecules for targeted application, not efficiently realizing the emergence of unavoidable side effects and the loss of quality of life. Furthermore, synthetic molecules are relatively more expensive and often unaffordable for the common population. Some of the commonly quoted examples are bevacizumab and ipilimumab, which are priced at $50,000 and $120,000 per treatment episode, respectively [37]. In these premises, the use of natural molecules that also have additional merits (more tolerable, easily available, and economic) has been proposed [38]. Several molecules of natural origin have been investigated for their anticancer potential. Fucoxanthin has previously been shown to demonstrate robust biological responses, including growth arrest [3] and apoptosis [7] at higher doses, and glial differentiation [16] and migration inhibition [12] at relatively non-toxic doses. In the present study, we sought to dissect the anti-metastatic potential of fucoxanthin.

Our results show that fucoxanthin could dock and form stable interactions at the mortalin binding site of p53, thereby preventing their complex formation (Figure 1A–C). As a result, it could activate wild-type p53 as confirmed by PG-13-Luc reporter assay (Figure 1F). Evolutionarily conserved p53 (wild type) plays a central role in cell growth and apoptotic pathways, metastasis checks, and dynamic central dogma [20,26]. It has been found to be inhibited by mortalin [27], enriched in large variety of cancers, and to contribute to carcinogenesis and metastasis. We found that fucoxanthin caused the abrogation of mortalin–p53 interactions, leading to the nuclear translocation of p53, and the reactivation of its transcriptional activation function in cancer cells. p53 mutant cell lines have been described to be more aggressive than their wild type or null counterpart because of the gain of functions that cause resistance against the stress and escalation of metastatic capabilities [26]. Bernardini et al. recently examined the cytotoxic effects of brown seaweed Padina pavonica extract in osteosarcoma cells with variable p53 status (p53 null Saos-2 and p53 mutant MNNG cells) [39]. The extract comprising mostly of fucosterol caused relatively higher cytotoxicity in p53 mutant-type cells than the p53 null cells, as seen in FITC Annexin V/Propidium Iodide assay. The finding was further supported by the stronger activation of pro-caspase-3 in mutant p53 cells. In the present study, we found that fucoxanthin, unlike fucosterol, was almost equally effective to treat cancer cells with wild type, mutant, as well as null p53 status (Figure S1A). Biochemical data on DLD-1 (harbor Ser to Phe mutation at the 241-amino acid residue of p53 [40]) endorsed that the subtoxic doses of fucoxanthin caused a remarkable delay in migration, which was well marked by the expression changes in proteins involved in cell proliferation, migration, and invasion (Figure 4 and Figure 5). Furthermore, we found that the effect of fucoxanthin was not dependent on p53, which was supported by the transcriptional repression of mortalin (Figure 3D) and the inhibition of its downstream signaling involved in cell migration and metastasis. Wang et al. [41] have also reported that fucoxanthin induces growth arrest and apoptosis by the downregulation of mortalin in human bladder cancer T24 cells that possess mutant p53.

In order to confirm the safety of fucoxanthin in in vivo conditions, we performed a relative hemolytic activity test at 37 °C (in vitro incubation of cells and in vivo pharmacodynamics) and found that fucoxanthin does not cause the hemolysis of erythrocytes (Figure S3). While establishing the doses, we observed that the discordance in the cytotoxic potential of fucoxanthin (comparing its activity against U2OS cells in Figure 2A,C) could be due to fucoxanthin’s chemical instability. In order to test the stability, we performed UV spectrophotometry analysis on fucoxanthin samples exposed to light and high-temperature conditions. As shown in Figure S2 and reported earlier [42], fucoxanthin showed sensitivity to light and heat, suggesting its chronometric degradation and hence the need for regular and more frequent intake for its pharmaceutical and therapeutic benefits. In this premise, our data indicating that the low doses of fucoxanthin possess strong anti-metastatic efficacy favors its use as natural anti-cancer drug, despite its low stability to light and heat. At the same time, studies on structural analogs with higher stability and efficacy, their bioactivities, and mechanism of action are warranted.

## 4. Materials and Methods

### 4.1. Cell Lines and Reagents

A549, DLD-1, H1299, MCF7, MDA-MB-231, MRC5, SKOV3, TIG-3, and U2OS cell lines were procured from JCRB, Japan, cultured in DMEM supplemented with 5% FBS and 1% penicillin/streptomycin (Invitrogen, Carlsbad, CA, USA) followed by incubation in a 37 °C incubator with 5% CO_2_ and 50% humidity. Stock concentration (5 mM) of fucoxanthin (Wako, Japan, 063-06691) was prepared in DMSO that was aliquoted and always stored at −20 °C in dark. For each experiment, a fresh aliquot of fucoxanthin was thawed. Primary antibodies raised against β-catenin (Santa Cruz, CA, USA, SC-7963), fibronectin (Santa Cruz, SC-52331), MMP2 (Santa Cruz, SC-10736), mortalin [43], p53 (Santa Cruz, SC-6243), pRB (Cell Signaling, 9307S), pSTAT3 (Cell Signaling, 9133S), RB (Cell Signaling, 9309S), STAT3 (Santa Cruz, SC-8019), survivin (Santa Cruz, SC-10811), VEGF (Santa Cruz, SC-507), vimentin (Santa Cruz, SC-6260), Wnt1 (Santa Cruz, SC-514531), and β-actin (AbCam, ab49900) were used in various probing assays. Secondary antibodies used in immunostaining were either Alexa-Fluor 488 goat anti-mouse IgG (Life Technologies, A11029, Carlsbad, CA, USA) or Alexa-Fluor 594 goat anti-rabbit IgG (Life Technologies, A11037). Secondary antibodies used in Western blotting were either goat anti-mouse HRP (Santa Cruz, sc-2005) or goat-anti-rabbit HRP (Santa Cruz, sc-2004). Recombinant mortalin protein was used to generate the standard curve in mortalin sandwich ELISA [44].

### 4.2. In Silico Assessment of Interaction of Fucoxanthin with Mortalin–P53 Complex

The possibility of the obstruction of mortalin–p53 complex formation by fucoxanthin was investigated using computational approaches of molecular docking and molecular dynamics simulation. The three-dimensional structure of p53 (PDB: 1OLG) and mortalin (PDB: 4KBO) was downloaded from the Protein Data Bank [45,46] and the structure of fucoxanthin (CID: 5281239) was retrieved from the PubChem database. Schrödinger Maestro Suite (Biologics Suite 2018-3, Schrödinger, LLC, New York, NY, 2018), Glide 8.0, and Desmond 5.5 were used for preparing the proteins and ligand structures, docking, and molecular dynamics simulations, respectively [47,48].

Fucoxanthin was docked to the p53 binding site of mortalin (253–282 amino acids), as well as to the mortalin binding site of p53 (oligomerization domain residues 323–337) [49]. Grids were generated around these two binding sites in the corresponding proteins, and docking was performed using the XP glide docking protocol [50]. To ensure the stability of the interaction between the ligand and protein, docking was followed by molecular dynamics simulation. The docked complex was solvated in a TIP3P water model, and simulated in NPT ensemble for 100 nanoseconds at 300 K and 1 atm pressure.

### 4.3. Immunostaining

Cells (25,000/well) were plated on glass coverslips, placed in a 12-well plate, and allowed to settle overnight; then, they were incubated either in control (DMSO) or fucoxanthin-supplemented culture medium for 48 h followed by fixation in pre-chilled methanol: acetone (1:1) on ice for 5 min, as described earlier [51]. Fixed cells were permeabilized with TPBS (PBS with 0.2%Triton-X for 10 min), washed with PBS for 10 min, blocked with serum albumin (2% in TPBS for 60 min), and then incubated with respective primary antibodies, as indicated, overnight. Coverslips were extensively washed with TPBS (3 to 5 times, 10 min each) and then incubated with secondary antibodies for 2 h. After washing with TPBS (3 to 5 times, 10 mins each), coverslips were incubated with Hoechst 33342 (Invitrogen, H3570) for the nuclear staining of cells, washed with TPBS, mounted with Fluoromount™ Aqueous Mounting Medium (Sigma-Aldrich, F4680, St. Louis, MO, USA), and visualized under the microscope for fluorescence signal. Protein expression was quantified using ImageJ software (NIH, Bathesda, MD, USA) and plotted in percentage using Microsoft™ Office^©^ 2016 (Microsoft™, Redmond, WA, USA).

### 4.4. PG-13 Luciferase Reporter Assay

Cells (200,000/well) were plated in a 6-well plate and allowed to settle overnight. These cells were transfected with wild-type p53 responsive luciferase reporter plasmid (PG-13-Luc), as described earlier [27] using lipofectamine™ transfection vector reagent (Thermo Fisher 11668030, Waltham, MA, USA) following the manufacturer’s protocol. Transiently transfected cells were incubated either in control (DMSO) or fucoxanthin-supplemented culture medium for 48 h. Cells were then harvested, washed with PBS, lysed using passive lysis buffer (Promega, E1500, Madison, WI, USA), quantified for total protein concentration, and then mixed with luciferase assay substrate to measure luminescence by Tecan infinite M200^®^ Pro microplate reader (Tecan Group Ltd., Mannedorf, Switzerland) using a Luciferase Reporter kit (Promega, E1500) following the manufacturer’s protocol. Luciferase activity was quantified and plotted in percentage using Microsoft™ Office^©^ 2016.

### 4.5. Dose Titration

Cells (2000/well) were plated in a 96-well plate and allowed to settle overnight. These cells were cultured with varying concentrations of fucoxanthin for 48 h. Then, cytotoxicity was evaluated as previously described [51]. Cell images were taken using a bright field microscope at 40 to 100X magnifications. An MTT (3-(4,5-dimethylthiazol-2-yl)-2,5-diphenyltetrazolium bromide)-based viability assay was performed to quantify the results. First, 10 μL of MTT (Sigma-Aldrich, M2003-1G) in phosphate-buffered saline was added to each well, and incubated at same conditions for 3 to 5 h. The media and MTT from the wells were aspirated out and replaced with 100% DMSO, followed by the measurement of absorbance at 570 nm. Cell viability was calculated in percentage against the control to plot toxicity charts value using Microsoft™ Office^©^ 2016.

### 4.6. QCV Assay

Cells (500/well) were plated in a 6-well plate and allowed to settle overnight. This was followed by treatment with varying doses of fucoxanthin and incubation at 37 °C with 5% CO_2_. The fucoxanthin-supplemented medium was replaced every alternate day. After 10 to 20 days (when the cells had grown to 16 population doublings), cells were fixed in methanol:acetone (1:1) on ice for 5 min, stained with 0.5% crystal violet dye for 2 h, washed thoroughly, and left to dry overnight. Colony pictures were scanned, and cell photographs were taken under a microscope, followed by the dissolution of the dye and its quantification by absorbance measurement at 570 nm. Absolute cell count was quantified from the absorbance values using slope equations described previously [34]; afterwards, cells were fixed, stained, and de-stained into the solution.

### 4.7. Western Blotting

Cells (2 × 10^5^/well) were plated in a 6-well plate and allowed to settle overnight. This was followed by treatment of cells with varying doses of fucoxanthin. Control and treated cells were harvested after 48 h and analyzed for Western blotting, as previously described [50]. Band intensity was quantified using ImageJ software (NIH) and plotted in percentage using Microsoft™ Office^©^ 2016.

### 4.8. Mortalin ELISA

Cells (2 × 10^5^/well) were plated in a 6-well plate and allowed to settle overnight. This was followed by treatment of cells with varying doses of fucoxanthin. Control and treated cells were harvested after 24 h and analyzed for absolute mortalin concentration by sandwich ELISA as previously described [52], using Tecan infinite M200^®^ Pro microplate reader (Tecan Group Ltd., Mannedorf, Switzerland). Mortalin concentration was quantified and plotted in percentage using Microsoft™ Office^©^ 2016.

### 4.9. Reverse Transcriptase Polymerase Chain Reaction

Cells (2 × 10^5^/well) were plated in a 6-well plate allowed to settle overnight, followed by treatment with varying doses of fucoxanthin. Control and treated cells were harvested after 24 h, RNA was extracted, cDNA was synthesized, and amplified to quantify mortalin mRNA levels by RT-PCR, as previously described [16]. GAPDH was taken as an internal control. The used primers had sequences of FW-AGCTGGAATGGCCTTAGTCAT and RV-CAGGAGTTGGTAGTACCCAAATC for mortalin, and FW-TGGAAATCCCATCACCATCT and RV-TTCACACCCATGACGAACAT for GAPDH. Band intensity was quantified using ImageJ software (NIH) and plotted in percentage using Microsoft™ Office^©^ 2016.

### 4.10. Wound Scratch Migration Assay and Matrigel Invasion Assay

The anti-migration and anti-invasiveness potential of fucoxanthin was evaluated by wound scratch migration and Matrigel invasion assay, respectively, as described previously [52]. Photographs of the scratch and migrating cells were captured after 48 h. Cell migration and invasion graphs were plotted considering the gap thickness/stain absorbance in control as 100% using Microsoft™ Office^©^ 2016.

### 4.11. Hemolytic Activity

Blood samples from a healthy mice heart were diluted with diluent buffer (0.85% NaCl containing 10 mM of CaCl_2_) to obtain 2% Red blood cell suspension (150 µL), incubated with 50 µM, 100 µM, and 200 µM of freshly aliquoted fucoxanthin (75 µL) in a 37 °C incubator in the dark for 30 min with occasional gentle pipetting. Then, the mixture was centrifuged at room temperature at 16,000 rpm for 5 min. Afterward, 200 µL of the supernatant was loaded into a 96-well plate to read absorbance at 540 nm. The ratio of absorbance corresponded to the degree of hemolysis, and 0.2% Triton X-100 in diluent buffer was taken as a positive control. Relative hemolytic activity was calculated in percentage against the positive control to plot a graph using Microsoft™ Office^©^ 2016. Mice were treated ethically as per the ethical guidelines and recommendations of the Animal Experiment Committee, Safety and Environment Management Division, National Institute of Advanced Industrial Science and Technology (AIST), Japan (Experimental plan approval #2012-025).

### 4.12. UV Spectrophotometry

Fresh aliquots of fucoxanthin at the concentration of 5 mm in DMSO were stored at various conditions of temperature and light, for variable time periods (as indicated), after which they were diluted in DMSO at the ratio of 1:325. The final volume was gently pipetted and poured into cuvettes to read the UV absorption spectra of fucoxanthin in 200 to 800-nm range and identify the λ_max_ using UV Probe 2.43 software and UV-VIS spectrophotometer (Shimadzu^®^ Corporation, UV-2600, Kyoto, Japan). DMSO was taken as the reference. Absorbance spectra from 200 to 555 nm were plotted by scatter plots using Microsoft™ Office^©^ 2016.

### 4.13. Statistical Significance

All the quantifications were performed using ImageJ software (NIH); calculations were done and plotted in percentage using Microsoft™ Office^©^ 2016. Statistical significance was calculated by an unpaired t-test of GraphPad^®^ software (2018–2019) (GraphPad^®^, San Diago, CA, USA) using mean, SD (standard deviation), and N (number) from three independent experiments, and shown as * *p* <0.05, ** *p* < 0.01, *** *p* < 0.001 or ns = not significant.

## Supplementary Materials

The following are available online at https://www.mdpi.com/1660-3397/17/6/338/s1, Figure S1A: Table showing IC50 values of fucoxanthin in cell lines with variable p53 status from Figure 2C. Figure S1B: QCV assay analysis of DLD-1 cells treated with fucoxanthin for 8 days continually for long-term cell viability and clonogenicity. Figure S2: A. Scatter plot from the UV-spectrometry for 10 µg/mL fucoxanthin (λmax = 446 nm) showing its optimum storage condition at −20 °C with degradation at higher temperatures. B. Scatter plot from the UV-spectrometry for 10 µg/mL fucoxanthin showing that its activity at 37 °C diminishes with increase in incubation time. Figure S3: Hemolytic activity plot showing that fucoxanthin caused insignificant hemolysis at 37 °C up to 200 µM.
