# Supplementary figures and images for: Marine Carotenoid Fucoxanthin Possesses Anti-Metastasis Activity: Molecular Evidence
Source: Mar Drugs. 2019 Jun 5;17(6):338. doi: 10.3390/md17060338 (PMC6627158; doi:10.3390/md17060338)

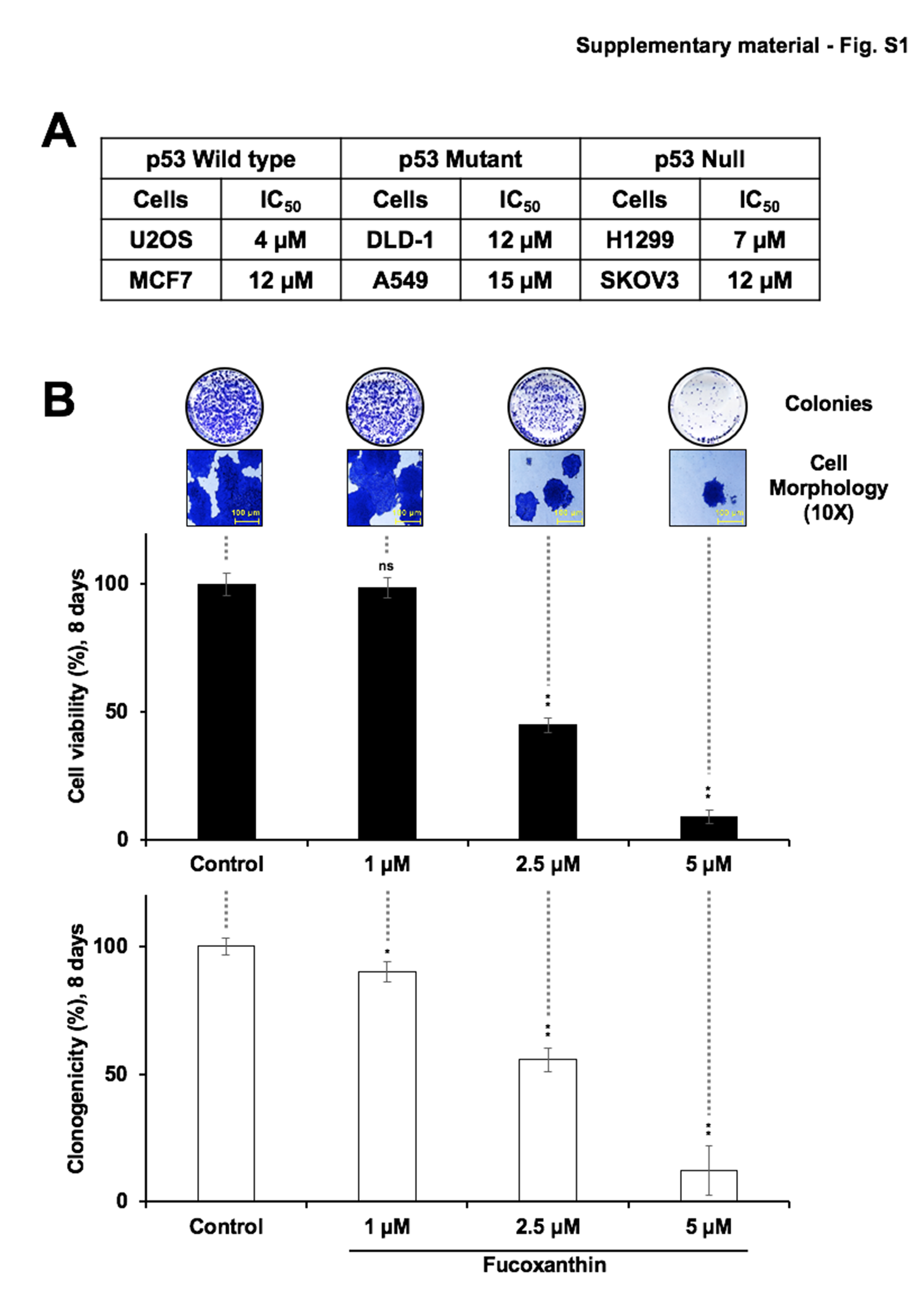

Supplement: Supplementary file 1 [file marinedrugs-17-00338-s001.zip › Figure S1.tif]

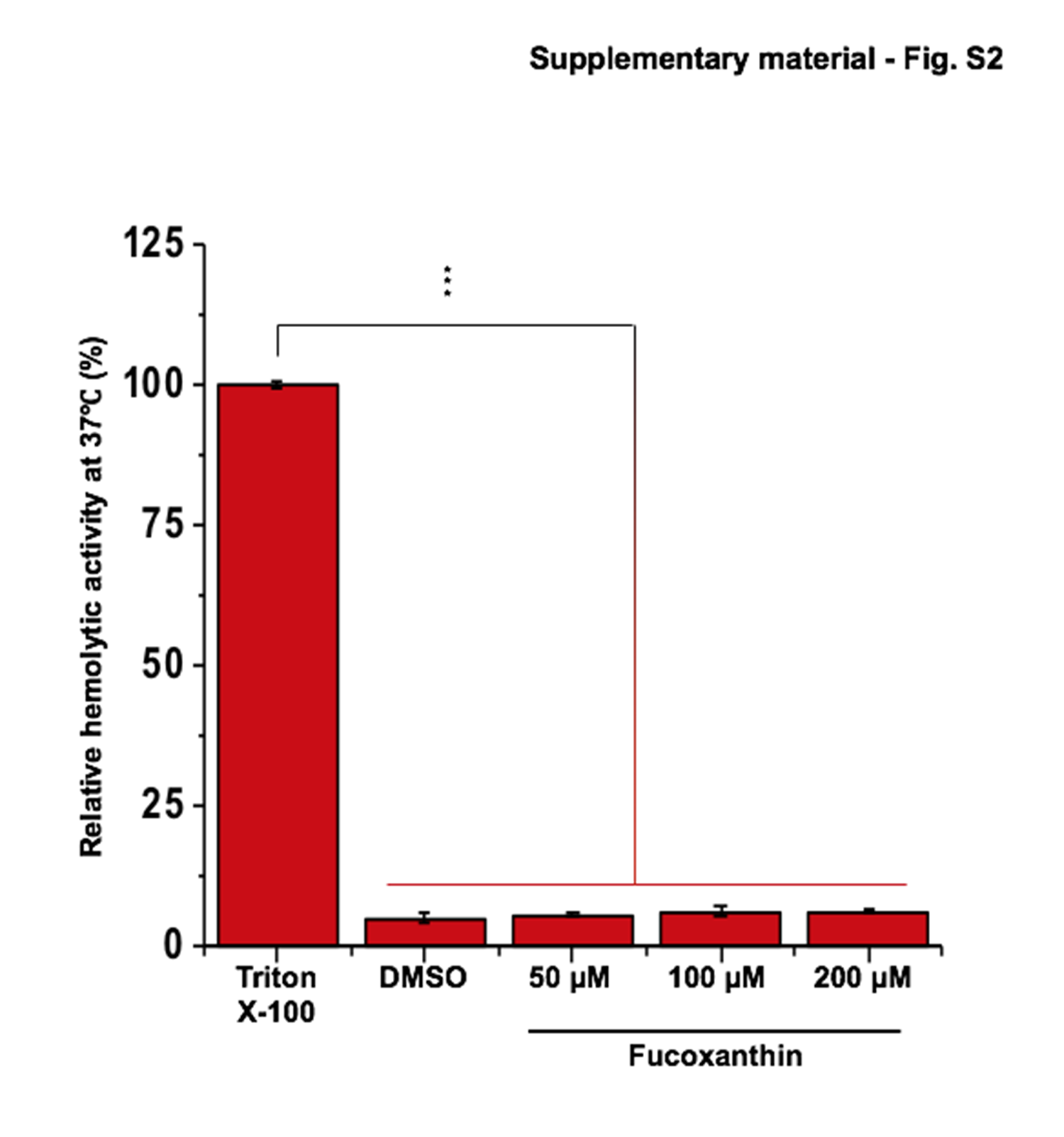

Supplement: Supplementary file 1 [file marinedrugs-17-00338-s001.zip › Figure S2.tif]

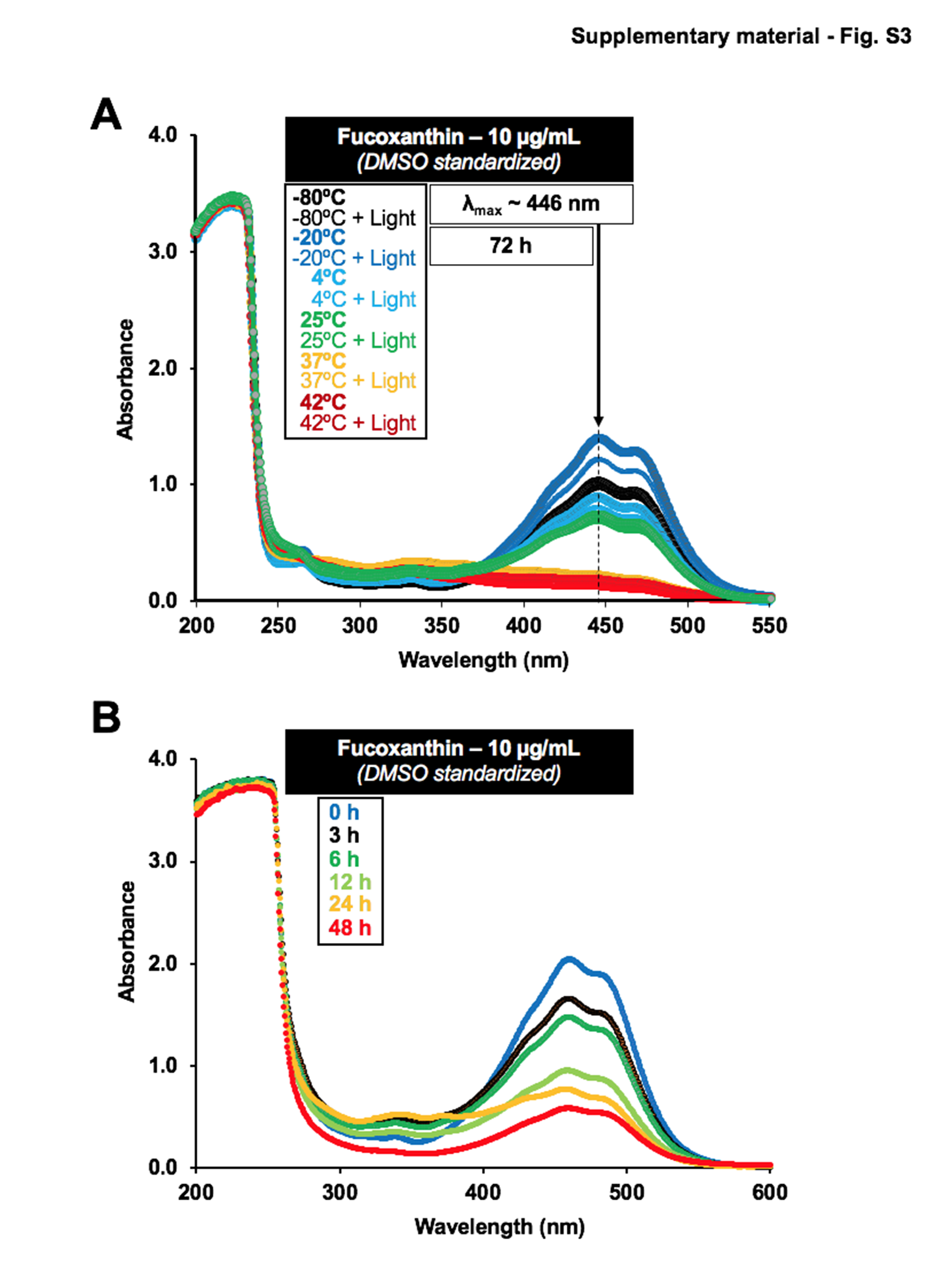

Supplement: Supplementary file 1 [file marinedrugs-17-00338-s001.zip › Figure S3.tif]
